# Germline mutations in *WNK2* could be associated with serrated polyposis syndrome

**DOI:** 10.1136/jmg-2022-108684

**Published:** 2022-10-21

**Authors:** Yasmin Soares de Lima, Coral Arnau-Collell, Jenifer Muñoz, Cristina Herrera-Pariente, Leticia Moreira, Teresa Ocaña, Marcos Díaz-Gay, Sebastià Franch-Expósito, Miriam Cuatrecasas, Sabela Carballal, Anael Lopez-Novo, Lorena Moreno, Guerau Fernàndez, Aranzazu Díaz de Bustamante, Sophia Peters, Anna K Sommer, Isabel Spier, Iris B A W te Paske, Yasmijn J van Herwaarden, Antoni Castells, Luis Bujanda, Gabriel Capellà, Verena Steinke-Lange, Khalid Mahmood, JiHoon Eric Joo, Julie Arnold, Susan Parry, Finlay A Macrae, Ingrid M Winship, Christophe Rosty, Joaquin Cubiella, Daniel Rodríguez-Alcalde, Elke Holinski-Feder, Richarda de Voer, Daniel D Buchanan, Stefan Aretz, Clara Ruiz-Ponte, Laura Valle, Francesc Balaguer, Laia Bonjoch, Sergi Castellvi-Bel

**Affiliations:** 1 Department of Gastroenterology, Institut d’Investigacions Biomèdiques August Pi i Sunyer (IDIBAPS), Centro de Investigación Biomédica en Red de Enfermedades Hepáticas y Digestivas (CIBERehd), Hospital Clínic, Barcelona, Spain; 2 Department of Cellular and Molecular Medicine, University of California San Diego (UCSD), San Diego, CA, USA; 3 Department of Pathology, Memorial Sloan Kettering Cancer Center, New York, NY, USA; 4 Department of Pathology, Hospital Clinic de Barcelona, Institut d’Investigacions Biomèdiques August Pi i Sunyer (IDIBAPS), Centro de Investigación Biomédica en Red de Enfermedades Hepáticas y Digestivas (CIBERehd) and Tumor Bank-Biobank, Barcelona, Spain; 5 Fundación Publica Galega de Medicina Xenómica (FPGMX), Grupo de Medicina Xenómica-USC, Instituto de Investigación Sanitaria de Santiago (IDIS), Centro de Investigación Biomédica en Red de Enfermedades Raras (CIBERER), Santiago de Compostela, Spain; 6 Department of Genetic and Molecular Medicine-IPER, Hospital Sant Joan de Déu and Institut de Recerca Sant Joan de Déu, Center for Biomedical Research Network on Rare Diseases (CIBERER), Barcelona, Spain; 7 Department of Genetics, Hospital Universitario de Mostoles, Mostoles, Spain; 8 Institute of Human Genetics, Medical Faculty, University of Bonn, Bonn, Germany; 9 National Center for Hereditary Tumor Syndromes, University Hospital Bonn, Bonn, Germany; 10 Department of Human Genetics, Radboud Institute for Molecular Life Sciences, Radboud University Medical Center, Nijmegen, The Netherlands; 11 Department of Gastroenterology and Hepatology, Radboud University Medical Centre, Nijmegen, The Netherlands; 12 Gastroenterology Department, Hospital Donostia-Instituto Biodonostia, Centro de Investigación Biomédica en Red de Enfermedades Hepáticas y Digestivas (CIBERehd), Basque Country University (UPV/EHU), San Sebastian, Spain; 13 Hereditary Cancer Program, Institute of Oncology, Oncobell, Institut d'Investigació Biomèdica de Bellvitge (IDIBELL), Centro de Investigación Biomédica en Red de Cáncer (CIBERONC), L'Hospitalet de Llobregat, Barcelona, Spain; 14 Medizinische Klinik und Poliklinik IV, Campus Innenstadt, Klinikum der Universität München, Munich, Germany; 15 MGZ – Center of Medical Genetics Center, Munich, Germany; 16 Colorectal Oncogenomics Group, Department of Clinical Pathology, Melbourne Medical School, The University of Melbourne, Parkville, Victoria, Australia; 17 University of Melbourne Centre for Cancer Research, The University of Melbourne, Parkville, Victoria, Australia; 18 Melbourne Bioinformatics, The University of Melbourne, Carlton, Victoria, Australia; 19 New Zealand Familial Gastrointestinal Cancer Service, Auckland, New Zealand; 20 Colorectal Medicine and Genetics, Royal Melbourne Hospital, Parkville, Victoria, Australia; 21 Genomic Medicine and Family Cancer Clinic, Royal Melbourne Hospital, Parkville, Victoria, Australia; 22 Department of Medicine, The University of Melbourne, Parkville, Victoria, Australia; 23 Envoi Specialist Pathologists, Brisbane, Queensland, Australia; 24 University of Queensland, Brisbane, Queensland, Australia; 25 Gastroenterology Department, Complexo Hospitalario Universitario de Ourense, Instituto de Investigación Sanitaria Galicia Sur, Centro de Investigación Biomédica en Red de Enfermedades Hepáticas y Digestivas (CIBERehd), Ourense, Spain; 26 Digestive Disease Section, Hospital Universitario de Móstoles, Móstoles, Spain

**Keywords:** Genetic Predisposition to Disease, Gene Editing, Gastroenterology, Loss of Function Mutation, Digestive System Disease

## Abstract

**Background:**

Patients with serrated polyposis syndrome (SPS) have multiple and/or large serrated colonic polyps and higher risk for colorectal cancer. SPS inherited genetic basis is mostly unknown. We aimed to identify new germline predisposition factors for SPS by functionally evaluating a candidate gene and replicating it in additional SPS cohorts.

**Methods:**

After a previous whole-exome sequencing in 39 SPS patients from 16 families (discovery cohort), we sequenced specific genes in an independent validation cohort of 211 unrelated SPS cases. Additional external replication was also available in 297 SPS cases. The *WNK2* gene was disrupted in HT-29 cells by gene editing, and *WNK2* variants were transfected using a lentiviral delivery system. Cells were analysed by immunoblots, real-time PCR and functional assays monitoring the mitogen-activated protein kinase (MAPK) pathway, cell cycle progression, survival and adhesion.

**Results:**

We identified 2 rare germline variants in the *WNK2* gene in the discovery cohort, 3 additional variants in the validation cohort and 10 other variants in the external cohorts. Variants c.2105C>T (p.Pro702Leu), c.4820C>T (p.Ala1607Val) and c.6157G>A (p.Val2053Ile) were functionally characterised, displaying higher levels of phospho-PAK1/2, phospho-ERK1/2, CCND1, clonogenic capacity and MMP2.

**Conclusion:**

After whole-exome sequencing in SPS cases with familial aggregation and replication of results in additional cohorts, we identified rare germline variants in the *WNK2* gene. Functional studies suggested germline *WNK2* variants affect protein function in the context of the MAPK pathway, a molecular hallmark in this disease.

**WHAT IS ALREADY KNOWN ON THIS TOPIC**

- Serrated polyposis syndrome (SPS) predisposes to colorectal cancer, and its inherited genetic basis is mostly unknown.

**WHAT THIS STUDY ADDS**

- Germline *WNK2* variants altering the MAPK pathway, a hallmark of this disease, could predispose to SPS.

**HOW THIS STUDY MIGHT AFFECT RESEARCH, PRACTICE OR POLICY**

- The identification of a novel hereditary SPS factor would permit a more accurate diagnosis of SPS patients and facilitate genetic counselling and prevention.

## Introduction

Colorectal cancer (CRC) is one of the most common cancers worldwide with a significant associated mortality. Aside from lung cancer, with an avoidable environmental cause, CRC is responsible for more deaths than any other malignancy in Western countries.^1^ The vast majority of CRC cases develop through an adenoma-carcinoma sequence.^2^ In recent years, another carcinogenesis pathway has been identified: the serrated pathway, starting from a different precancerous lesion, the serrated polyp. Although serrated polyps were previously considered indolent, current evidence estimates they are the precursor lesion for up to 30% of CRC cases.^3^

Serrated polyposis syndrome (SPS) is a clinical condition characterised by the presence of multiple and/or large serrated polyps in the colon, as well as an associated higher risk of CRC.^4 5^ The following criteria were established by the WHO in 2010 in order to help identifying SPS patients: (1) at least five serrated lesions/polyps proximal to the sigmoid colon with two or more of these being >10 mm, (2) any number of serrated polyps proximal to the sigmoid colon in an individual who has a first-degree relative with serrated polyposis and (3) >20 serrated polyps of any size but distributed throughout the colon.^6^ This arbitrary definition is not based on any genetic alteration and has been considered somehow restrictive, leading to underdiagnosis of this syndrome. Recently, it was updated to not include the second criterion. Also, new criterion I includes polyps proximal to the rectum and polyps now have to be ≥5 mm. The updated criterion II now explicitly states that ≥5 of the serrated polyps should be located proximal to the rectum.^7^ Although its prevalence in the population is unknown, it could be higher than expected according to data from CRC screening.^8–11^ It is also probably underrecognised due insufficient knowledge in the medical community, the difficult endoscopic detection of serrated lesions/polyps (small size and flat morphology), the lack of understanding regarding germline predisposition and the absence of associated symptoms.

CRC, including those cases associated with SPS, as for other complex diseases, are caused by both genetic and environmental factors.^12^ Smoking, body mass index and alcohol intake have been highlighted as important environmental risk factors for serrated polyps.^13^ Importantly, twin studies also showed that around 13%–30% of the variation in CRC susceptibility involves inherited genetic factors.^14^ *APC*, *MUTYH* and the mismatch repair (MMR) genes are among the most relevant genes involved in the main forms of hereditary CRC, familial adenomatous polyposis, *MUTYH*-associated polyposis (MAP) and Lynch syndrome.^15^ However, SPS is a disease with mostly unknown inherited genetic basis compared with other gastrointestinal polyposis syndromes. It has also been advocated that SPS may not be hereditary but mostly environmental. However, familial clustering and a high CRC risk for first-degree relatives of SPS patients have been described, which supports the involvement of germline predisposition in a subset of cases.^16^

First reported in 2014, germline loss-of-function variants in *RNF43* were associated with the development of multiple sessile serrated adenomas.^17^ *RNF43*, as regulator of the DNA damage response and negative regulator of Wnt signalling, was considered a likely SPS susceptibility gene. Subsequent studies have highlighted that *RNF43* accounts for a small proportion (<3%) of the germline predisposition in SPS.^18 19^ Among the list of other genes reported so far to be potentially involved in SPS germline predisposition, *MUTYH* was reported in very few cases although its role in SPS is probably not relevant.^20 21^ Other genes involved in polyposis predisposition such as *BMPR1A, SMAD4, PTEN* and *GREM1* have been also screened with negative results.^22^

Additional efforts have been undertaken to identify candidate genes for germline predisposition to SPS. Our research group has postulated further germline candidates for SPS by using combined whole-exome sequencing (WES) and linkage studies in families with multiple members affected by SPS^23^ and by performing germline and somatic WES in 39 patients from 16 SPS families showing familial aggregation mainly compatible with an autosomal dominant pattern of inheritance.^24^ This last study highlighted *ANXA10*, *ASXL1*, *CFTR*, *DOT1L*, *HIC1*, *INO80*, *KLF3*, *MCM3AP*, *MCM8*, *PDLIM2*, *POLD1*, *TP53BP1*, *WNK2* and *WRN* as candidate genes for SPS germline predisposition.

Accordingly, the main objectives of this study were the identification of novel germline causal genes for SPS predisposition by replicating a candidate gene in independent SPS cohorts and performing a functional evaluation of the detected rare variants. Confirmation of germline predisposition to SPS would permit a more accurate and adequate diagnosis of patients, as well as facilitating genetic counselling and prevention.

## Materials and methods

### Patients

The discovery cohort comprised 39 patients from 16 families (≥2 patients per family) diagnosed with SPS and fulfilling the 2010 WHO criteria.^6^ The updated 2019 WHO criteria were not available when this study was initiated and developed for the discovery cohort.^7^ A complete clinical characterisation of this discovery cohort was previously published.^24^ No patients in the discovery cohort presented with pathogenic variants in *MUTYH*, *APC* or the DNA MMR genes, when analysed using gene panel sequencing and screening for point mutation, copy-number variants and potential splicing alterations.

Two hundred and eleven unrelated Spanish SPS patients were recruited in high-risk CRC clinics at Hospital Clínic de Barcelona, Institut Català d’Oncologia-IDIBELL and Fundación Pública Galega de Medicina Xenómica and were used as validation cohort. Additional external SPS cohorts (n=297) with available sequencing data for unrelated patients from the University of Bonn and the Medical Genetics Center Munich in Germany (n=168), the Radboud University Medical Centre in the Netherlands (n=29) and the Genetics of Colonic Polyposis Study from Australia and New Zealand (n=100) were also accessed. The 2010 WHO criteria were also used in this cohort for consistency.

### Variant identification

For more details on variant identification and validation, see online supplemental material.

#### Variant prioritisation

Variant prioritisation was carried out considering several aspects. First, we only took into consideration those variants present in the canonical transcripts. Also, dominant and recessive analysis were pursued. Homozygous/compound heterozygous variants in relevant genes were not identified. Therefore, only heterozygous variants were further considered. In addition, a minimum allele frequency of 0.1% was required for variant filtering and only non-synonymous and/or truncating variants were prioritised. The missense variants had to fulfil at least three out of six pathogenic predictions used for analysis (PhyloP, SIFT, Polyphen, MutationTaster, CADD and LRT). The next crucial step of variant prioritisation considered data integration with the first cohort results. We prioritised genes that presented germline variants in both cohorts and conducted an extensive literature research over possible connections between candidate genes and SPS. Only candidate genes with rare, nonsynonymous/truncating or missense variants fulfilling at least three out of six pathogenic predictions detected in the discovery and the validation cohort were further considered. Among them, only those with a function compatible with SPS, CRC or cancer were selected.

#### Gene panel sequencing

For the validation cohort, a panel of 20 genes was designed using the DesignStudio online tool (Illumina, San Diego, USA). We included 14 genes suggested as plausible candidates to SPS in the discovery cohort comprising *ANXA10*, *ASXL1*, *CFTR*, *DOT1L*, *HIC1*, *INO80*, *KLF3*, *MCM3AP*, *MCM8*, *PDLIM2*, *POLD1*, *TP53BP1*, *WNK2* and *WRN*.^24^ Additional genes were also included, such as *ANXA1* and *ANXA2* (histological markers for SPS, same family as *ANXA10*), *ASLX2* (same gene family as *ASXL1*), *POLE*, *FBLN2* (previously suggested by our group)^23^ and *RNF43*, previously involved in germline SPS predisposition.

#### Gene panel analysis

The raw sequencing data were first analysed using the Miseq Reporter software (Illumina, San Diego, USA). First, the data were aligned to the hg19 human genome using the Burrows-Wheeler Aligner (BWA-MEM).^25^ Then, variant calling was conducted using the Germline Variant Caller (Illumina, San Diego, USA). Variant annotation was performed as previously described using SnpEff and SnpSift software (https://pcingola.github.io/SnpEff/).^24 26^

### Functional characterisation of genetic variants

For details on the development of a cellular model for variant characterisation, ERK1/2 and PAK1 assays, see online supplemental material. All plasmids, antibodies, restriction enzymes and Taqman probes used in this study are listed in online supplemental table 1. Primer details are listed in online supplemental table 2. If not indicated otherwise, functional assays were developed with HT-29 cells cultured in McCoy 5A media supplemented with 5% FBS and 1 µg/mL of doxycycline.

10.1136/jmg-2022-108684.supp1 Supplementary data

#### MAPK pathway activity: ERK1/2 and PAK1 phosphorylation

To detect total phospho-ERK1/2, cells were stimulated with 1 ng/mL human epidermal growth factor (hEGF) for 10 min and assayed with the Phospho-ERK1 (T202/Y204)/ERK2 (T185/Y187) DuoSet IC ELISA kit according to the manufacturer’s protocol (Bio-Techne, Minnesota, USA). Phospho-ERK1/2 levels between EGF-stimulated and non-stimulated conditions were quantified.

To detect phosphorylated PAK1, an In-Cell ELISA assay was done. The 96-well plates were coated with 50 µg/mL Poly-L-Lysine before cell seeding. Cells were stimulated with 10 ng/mL of hEGF for 5 min, immediately fixed and assayed with phospho-PAK1 (rabbit) and β-actin (mouse) antibodies overnight at 4°C. The multiplexed detection of both targets was performed with the antimouse Dylight 800 (ThermoFisher, Waltham, Massachusetts, USA) and antirabbit IRDye 680RD (LI-COR, Lincoln, Nebraska, USA) antibodies. Plates were scanned with Odyssey (LI-COR) and analysed with Image Studio 4.0 software.

#### *CCND1* and *MMP2* expression

Cells were seeded in P60 dishes at 600 000 cells per plate and left to grow for 2 days. Then, cells were stimulated with 1 ng/mL of hEGF for 16 hours in serum-free McCoy 5A media with 1 µg/mL of doxycycline. The next day, cells were detached using a cell-scrapper, and the RNA was extracted using the RNeasyMini Kit according to the manufacturer’s protocol (Qiagen, Hilden, Germany). Retrotranscription and quantitative PCR were done as described in online supplemental material.

#### Clonogenic assay

Cells were seeded at low density, at 200 cells per well in a 6-well plate. Cells were maintained either in the presence of 1 ng/mL of hEGF or without hEGF. After 16 days, cells were fixed with methanol for 10 min and stained with a 0.5% crystal violet solution.

#### Adhesion assay

Before cell seeding, 96-well plates were coated with 5 µg/mL fibronectin in PBS 1X and incubated for 1 hour at room temperature. Next, the plate was dried out and blocked with BSA 1% for an additional hour. A total of 40 000 cells were seeded per well and left to attach for 60 min. Subsequently, unattached cells were removed by inversion; the plate was washed carefully with serum-free McCoy 5A and fixed with methanol for 10 min. Finally, fixed cells were stained with a 0.5% crystal violet solution. Images were captured on an AID EliSpot reader system and analysed with the ReadPlate 3.0 plugin for Image J.

## Results

After variant prioritisation, only candidate genes with rare, non-synonymous/truncating or missense variants fulfilling at least three out of six pathogenic predictions detected in the discovery and the validation cohort were further considered. The WNK lysine deficient protein kinase 2 (*WNK2*) gene stood out among others for being a negative regulator of the mitogen-activated protein kinase (MAPK) pathway.^27^ MAPK cascades are central signalling pathways that regulate basic processes, including cell proliferation, differentiation, stress responses and apoptosis. Mutations in these pathways lead to their constitutive activation and uncontrolled cell proliferation.^28^ One of the cascades, MAPK/ERK, is of particular interest in SPS because one of its components, *BRAF*, shows activating mutations in approximately 75% of sessile serrated polyps.^29^ Due to its role as a negative regulator of this pathway, *WNK2* was considered a promising candidate gene for germline SPS predisposition.

**Figure 1:**
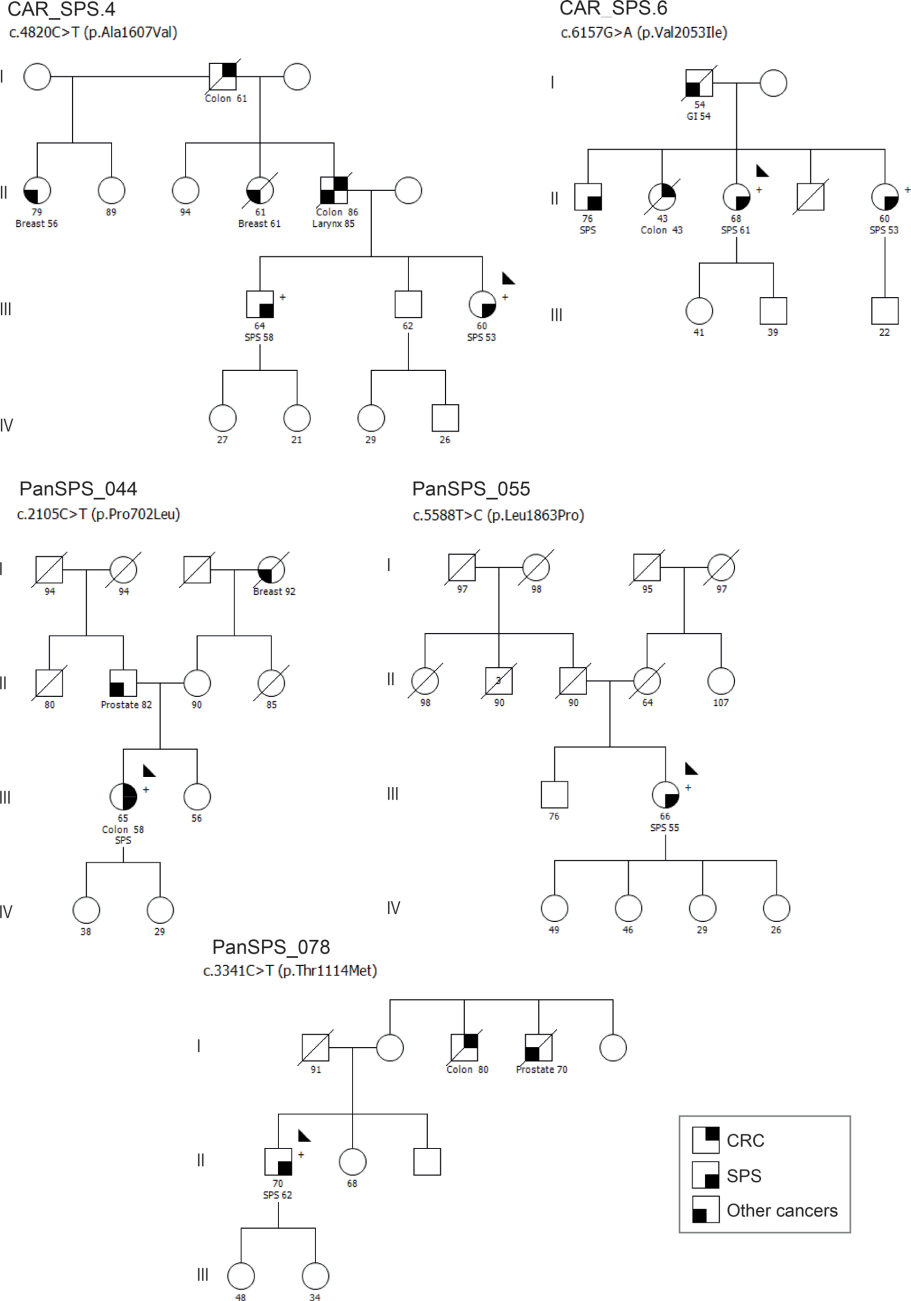
Pedigrees of five SPS families. Filled symbol indicates affected for CRC (upper right quarter), SPS (lower right quarter) or other types of cancer (lower left quarter). CRC, colon, breast, larynx, GI (gastrointestinal) and prostate refer to the type of cancer. Ages at diagnosis are depicted. The proband is indicated by an arrow. Variant carriers are indicated by (+). CRC, colorectal cancer; SPS, serrated polyposis syndrome.

In the discovery cohort, two *WNK2* variants were detected including c.4820C>T (p.Ala1607Val) in family CAR_SPS.4, and c.6157G>A (p.Val2053Ile) in family CAR_SPS.6 (figure 1). These variants were classified as potentially damaging by five out of six missense pathogenicity prediction tools. Families showed CRC family history, and variants were detected in two family members affected with SPS, although no additional segregation analysis was possible. MMR system was preserved in the analysed serrated lesions from both families and the CAR_SPS.6 polyp was *BRAF* mutated. Loss of heterozygosity seeking a potential deletion of the wild-type (WT) *WNK2* allele was not detected in the analysed serrated lesions. We also performed WES on the most advanced serrated lesion available in one individual from each family, which allowed performing somatic mutational profiles. The single-base substitution (SBS) signatures SBS.1 and SBS.5, considered clock-like mutational signatures, were the most represented in both samples, and no other distinctive signature was apparent.^24^

In a validation SPS cohort of 211 unrelated patients, gene panel sequencing revealed four additional rare, missense variants in *WNK2* including c.2105C>T (p.Pro702Leu) in PanSPS_044, c.2792C>T (p.Thr931Met) in PanSPS_095, c.3341C>T (p.Thr1114Met) in PanSPS_078 and c.5588T>C (p.Leu1863Pro) in PanSPS_055. The c.2792C>T variant was detected in a male SPS patient (onset at 67 years) in a family where an additional sample for variant segregation was available. It corresponded to a sister of the proband with CRC (72 y.o.). However, this variant was finally excluded for further studies since segregation was not confirmed. The tumour sample in PanSPS_044 (III-1) showed loss of expression for MLH1/PMS2 and was *BRAF* mutated and *MGMT* methylated. No similar information was available regarding MMR system or somatic alterations for any serrated lesion from PanSPS_055 and PanSPS_078. Pedigrees and the five *WNK2* variants that were selected are summarised in figure 1 and table 1.

**Table 1:** Rare germline variants identified in the *WNK2* gene in the discovery and validation SPS cohorts

Variant	Exon	Prediction tools	gnomAD	Allele count	Number of homzygotes	Family	SPS criteria	CRC index case	CRC family history	Cohort
c.2105C>T (p.Pro702Leu)	9	5	0.000125	19/152,210	0	PanSPS_044	3	Y	N	Validation
c.3341C>T (p.Thr1114Met)	12	3	0.0000526	8/152,182	0	PanSPS_078	1	N	Y	Validation
c.4820C>T (p.Ala1607Val)	20	5	0.000138	21/152,228	0	CAR_SPS.4	1,3	N	Y	Discovery
c.5588T>C (p.Leu1863Pro)	23	5	0	0	0	PanSPS_055	3	N	N	Validation
c.6157G>A (p.Val2053Ile)	25	5	0.0000657	10/152,200	0	CAR_SPS.6	2,3	N	Y	Discovery

Prediction tools: number of pathogenicity positive predictions according to missense bioinformatics prediction tools (PhyloP, SIFT, Polyphen, MutationTaster, CADD and LRT). SPS criteria: according to the 2010 WHO SPS clinical criteria. CRC family history: defined as presence of any CRC case in the family besides the index case.

CRC, colorectal cancer; gnomAD, Genome Aggregation Database; N, no; SPS, serrated polyposis syndrome; Y, yes.

### Functional characterisation of *WNK2* depletion

To unequivocally assess the functional effect of the previous candidate variants and assess their link to SPS, it was important to reduce the masking effect of the endogenous WT *WNK2* expression in the selected cellular model. For this reason, we performed a two-step genetic engineering strategy. First, we knocked-out *WNK2* in the human cell line HT-29 by CRISPR-Cas9. Then, we reintroduced each of the *WNK2* variants of interest using a lentiviral delivery system, and specific functional studies were carried out. We confirmed the CRISPR-mediated *WNK2* gene editing by Sanger sequencing and selected two clones (*WNK2* ^KO2^ and *WNK2* ^KO7^) with no expression of WNK2 at both mRNA and protein levels (online supplemental figure 1).

**Figure 2:**
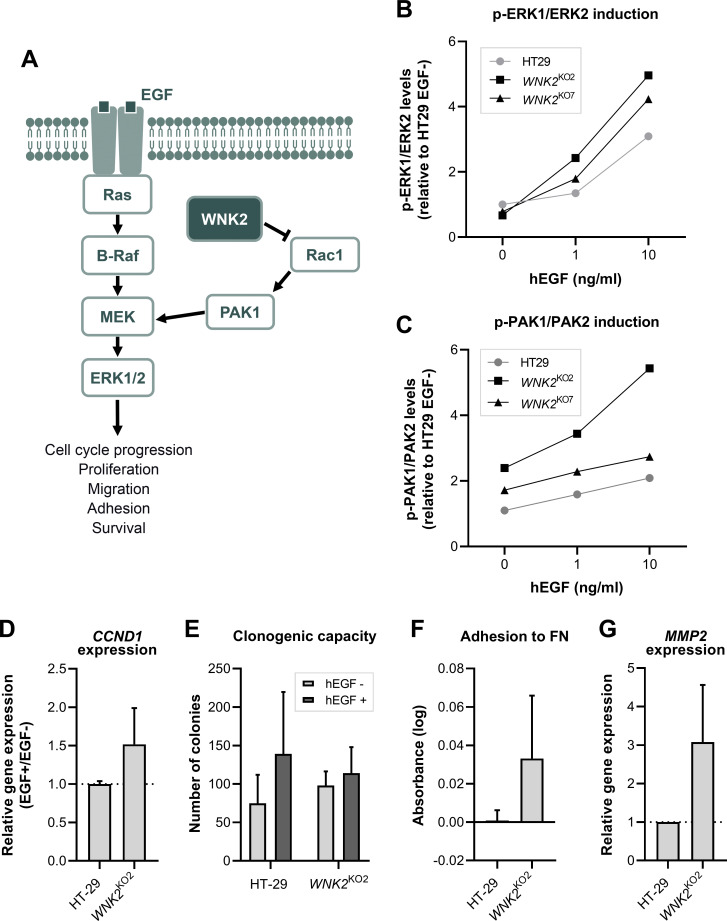
Functional characterisation of *WNK2* ^KO^ clones. (A) Diagram depicting the role of WNK2 in the ERK pathway. WNK2 affects GTP loading of Rac1, interfering in the cascade of the ERK signal transduction pathway. Adapted from Moniz and Jordan.^32^ (B) Dose-response of hEGF-induced ERK1/2 and (C) PAK1/2 phosphorylation levels in both *WNK2* ^KO2^ and *WNK2* ^KO7^ clones. Samples were assayed in triplicate. (D) *CCND1* mRNA relative expression after treatment with 1 ng/mL of hEGF. Data are expressed as EGF+/EGF− ratio, and mean±SD is represented (n=3). (E) Clonogenic capacity of cells cultured during 16 days in the presence or absence of 1 ng/mL of hEGF. Data represent mean±SD (n=4). (F) Fibronectin (FN)-mediated cell adhesion assay using crystal violet staining. Data represent mean±SD (n=3). (G) *MMP2* mRNA relative expression after treatment with 1 ng/mL of hEGF. Data represent mean±SD (n=3). The experiments were performed in triplicate and repeated three or four times, as indicated in each case. hEGF, human epidermal growth factor.

Afterwards, we evaluated the phenotype of the selected *WNK2* ^KO^ clones. WNK2 is a negative regulator of the ERK1/2 MAPK signalling cascade, where growth factors trigger signal transduction through a series of sequential protein phosphorylations. Specifically, WNK2 modulates the Rac1/PAK1-mediated activation of ERK1/2 (figure 2A). Therefore, we assessed the phosphorylated status of both ERK1/2 and PAK1/2. Treatment with hEGF promoted a dose-dependent stimulation of these targets in HT-29 control cells. We observed that the lack of WNK2 facilitated the activation of this pathway, as both clones displayed a higher increase in the phosphorylation of ERK1/2 and PAK1/2 (figure 2B,C), even though HT-29 cells are *BRAF* mutated. The effect was even more outstanding in clone *WNK2* ^KO2^, which showed the highest ERK1/2 and PAK1/2 phosphorylation levels at all tested doses.

We further characterised *WNK2* ^KO2^ cells to determine the functional consequences of the alteration in the MAPK pathway. Since this pathway promotes cell cycle progression, we tested Cyclin D1 (*CCND1*) expression, one of the main PAK/ERK targets in mitogenic signalling. We detected increased *CCND1* expression levels (figure 2D) and a higher clonogenic capacity (figure 2E) of *WNK2* ^KO2^ cells in comparison with control cells after hEGF induction, indicating that WNK2 depletion altered cell cycle progression and cell proliferation.

The activation of the MAPK pathway promotes the expression of metalloproteinases, which degrade the extracellular matrix and are associated with cellular adhesion and a more aggressive phenotype. Specifically, *WNK2* has been described to negatively regulate two metalloproteinases, MMP2 and MMP9.^30^ Therefore, we also analysed the effects of WNK2 depletion on cell adhesion and matrix metalloproteinase-2 (*MMP2*) expression, for which WNK2 has already been described to work as a negative regulator. *WNK2* ^KO2^ exhibited increased fibronectin-mediated cell attachment (figure 2F) and higher *MMP2* expression levels (figure 2G). Altogether, these results show that deletion of WNK2 in HT29 cells alters the regulation of downstream mediators of the MAPK pathway, one of the main pathways that drive serrated tumorigenesis, suggesting that alterations on this gene may be associated with the development of the serrated polyposis phenotype.

### Functional characterisation of *WNK2* germline variants

Three *WNK2* candidate variants were selected to investigate their functional effect including p.Ala1607Val and p.Val2053Ile from the discovery cohort and p.Pro702Leu from the validation cohort (table 1). Families carrying these variants had the most severe clinical presentation including SPS aggregation or SPS and CRC in the index case. These variants were also classified as potentially damaging by five out of six pathogenicity prediction tools. These variants were designed by site-directed mutagenesis and individually reintroduced in both *WNK2* ^KO2^ and *WNK2* ^KO7^ clones. As a control, the WT *WNK2* sequence was also introduced in both clones to rescue the original phenotype. We selected the optimal doxycycline dose (1 µg/mL) to obtain successful gene expression levels for each of the introduced *WNK2* sequences. All *WNK2* variants were equally expressed at both RNA and protein levels (online supplemental figure 2).

**Figure 3:**
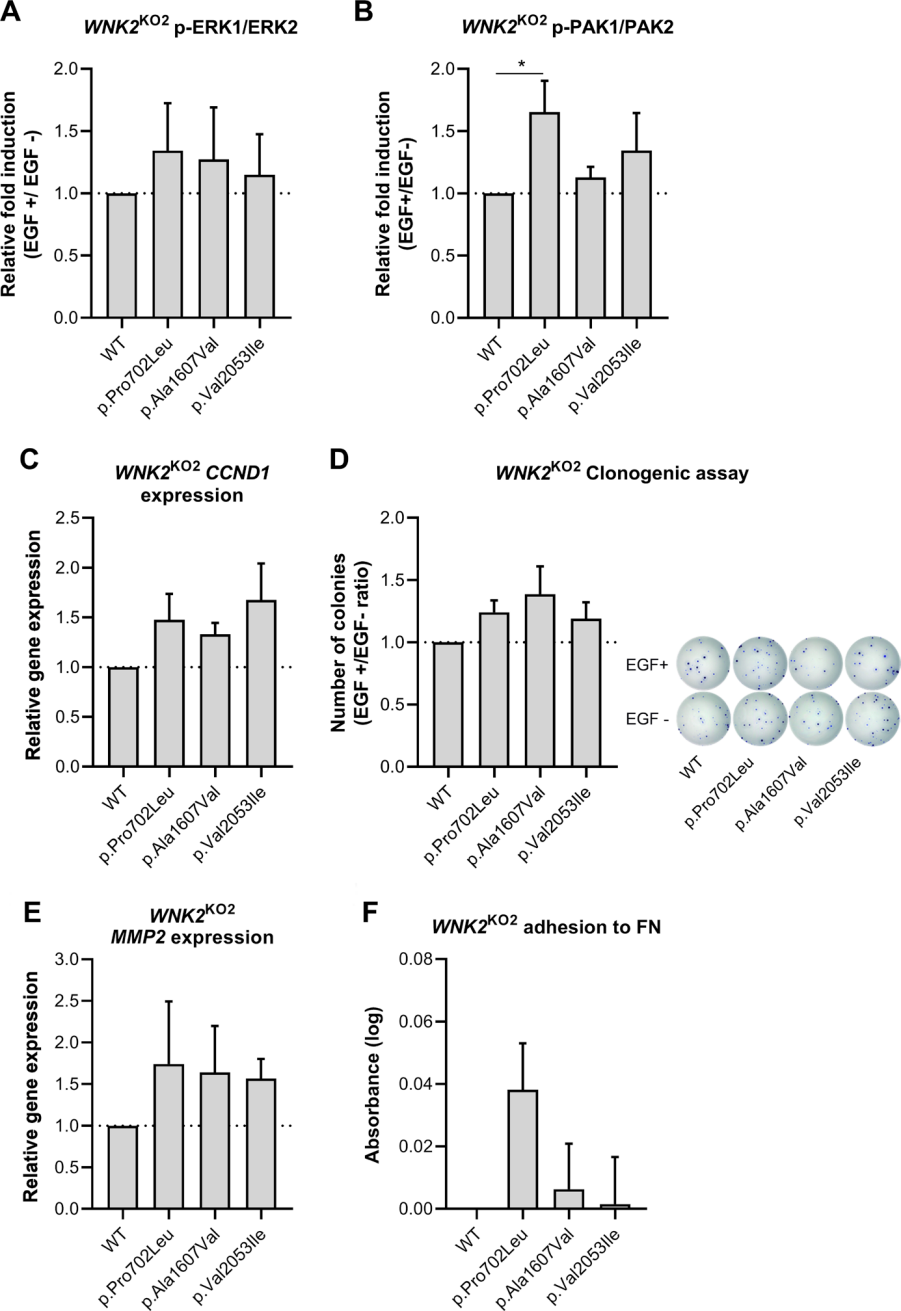
Variants in WNK2 activate the MAPK pathway and contribute to cell cycle deregulation, cell proliferation and altered cellular adhesion. *WNK2* ^KO2^ cells re-expressing either *WNK2* WT or each of the selected variants were functionally characterised. The variants were expressed successfully under the inducible promoter. The experiments were performed in triplicate and repeated three or four times, as indicated in each case. (A) ERK1/2 phosphorylation levels measured by ELISA in cell samples treated or untreated with 1 ng/mL hEGF. Data are displayed as EGF+/− ratio (n=3; mean±SD). (B) PAK1/2 phosphorylation levels measured by an In-Cell ELISA (ICE) assay in cell samples treated or untreated with 10 ng/mL hEGF. Data are displayed as EGF+/− ratio (n=3; mean±SD) (C) *CCND1* mRNA relative expression after a 16-hour treatment with 1 ng/mL of hEGF (n=3; mean±SD). (D) Clonogenic capacity of cells cultured during 16 days in the presence or absence of 1 ng/mL of hEGF. Data are displayed as EGF+/− ratio (n=4; mean±SD). On the right, representative images of methanol-fixed, crystal violet stained colonies. *P<0.05, analysis of variance with Fisher’s LSD post hoc test. (E) *MMP2* mRNA relative expression after a 16-hour treatment with 1 ng/mL of hEGF (n=3; mean±SD). The variants were expressed successfully under the inducible promoter. (F) *WNK2* ^KO2^ cells expressing either the WT sequence or each of the candidate variants were cultured on fibronectin (FN)-coated plates for 1 hour, and the adherent cells were detected by crystal violet staining (n=3; mean±SD). hEGF, human epidermal growth factor; WT, wild-type.

We then proceeded to do the functional characterisation of the selected variants. We first examined the phosphorylation status of ERK1/2 and PAK1/2, two key components of the MAPK pathway (figure 2A). *WNK2* ^KO2^ cells re-expressing either WNK2-WT or each of the selected variants were treated with hEGF, and both ERK1/2 and PAK1/2 phosphorylation levels were assessed. The activation of the pathway was determined before and after hEGF treatment (EGF+/− ratio). hEGF induced the phosphorylation of both ERK1/2 (figure 3A) and PAK1/2 (figure 3B) kinases. When comparing with the rescued WT *WNK2* phenotype, the MAPK activation was present to some extent for the three variants, being more noticeable with cells expressing the p.Pro702Leu variant.

Next, as the MAPK pathway promotes cell cycle progression, we tested whether *WNK2* variants would be implicated in *CCND1* expression. All missense *WNK2* variants promoted a moderate increase in *CCND1* expression in comparison with cells expressing the WT counterpart (figure 3C). A similar trend was observed when performing the clonogenic cell survival assay, in which cells expressing *WNK2* variants showed a higher survival rate (figure 3D).

Finally, we focused on cell adhesion and metalloproteinase *MMP2* expression. We first analysed the expression of *MMP2* after hEGF treatment. We observed a tendency for a higher expression of this marker for the three variants in comparison with cells expressing with WNK2-WT (figure 3E). Then, cells were subjected to an adhesion test to the extracellular matrix protein fibronectin, which is cleaved by MMP2. By doing so, we detected a greater adhesion capacity of cells expressing the p.Pro702Leu and p.Ala1607Val variants (figure 3F), compared with the rescued WT phenotype.

The functional characterisation of *WNK2* variants was replicated in clone *WNK2* ^KO7^ (online supplemental figure 3), focusing on the assays more directed to the MAPK pathway itself (ERK1/2 and PAK1/2), and similar results were obtained. Consistently, the activation of the MAPK pathway and the adhesion capacity of cells were higher when *WNK2* variants were expressed, again with a prominent effect in the case of variant p.Pro702Leu.

All in all, these results suggested that *WNK2* variants partially failed to repress the activation of this molecular pathway and were concordant in some measure with the alteration of the MAPK pathway, supporting the malfunctioning of *WNK2* variants.

### Screening of the candidate gene variants in additional SPS cohorts

International SPS cohorts from Germany, Australia and the Netherlands with available WES or whole-genome sequencing data were further consulted seeking for additional rare, non-synonymous/truncating or missense *WNK2* alterations in their patients (n=297). Genetic variants were selected based on low population frequency (<0.25%), deleterious effect on the protein and pathogenic bioinformatics prediction (Combined Annotation Dependent Depletion Phred (CADD) score >15). In summary, 10 additional rare, protein-altering *WNK2* genetic variants were identified in 12 SPS patients (table 2). Considering the number of variants found in all analysed SPS cohorts, the frequency of germline *WNK2* alterations in SPS could be considered ~3% (17/524, 3.24%).

**Table 2:** Rare, non-synonymous/truncating or missense variants in WNK2 identified in additional international SPS cohorts

Variant	Exon	gnomAD	Allele number	Number of homozygotes	CADD	Patient	SPS criteria	Onset age	CRC index case	Cohort
c.106_107insG (p.Pro36Argfs*121)	1	0	0	0	N/A	BN-174	1,3	22	N	DE
c.1853G>A (p.Ser618Asn)	8	0	0	0	26.4	RB-1	1	57	N	NL
c.2758G>A (p.Ala920Thr)	11	0.0001315	20/152 056	0	15	GCP208001	1,3	27	N	AU
c.3418G>A (p.Gly1140Ser)	14	0	0	0	23.9	MUC-6	1,3	42	NA	DE
c.3623C>T (p.Thr1208Met)	15	0.000006582	1/151 930	0	26.6	MUC-4	*	30	N	DE
c.5476C>T (p.Arg1826Trp)	23	0	0	0	25.1	BN-210	3	55	N	DE
c.5656C>T (p.Arg1886Trp)	23	0.002233	219/152 194	1	21.2	RB-2	†	51	N	NL
						BN-145	3	29	NA	DE
						MUC-1116–01	1	20	N	DE
c.5906C>G (p.Pro1969Arg)	24	0	0	0	25.3	GCP038001	3	59	N	AU
c.6080C>G (p.Ala2027Gly)	25	0	0	0	25.1	BN-83	3	18	NA	DE
c.6512G>A (p.Ser2171Asn)	28	0	0	0	23	BN-104	1,3	21	NA	DE

A cut-off of 15 was used to selected possible pathogenic variants. SPS criteria: according to the 2010 WHO SPS clinical criteria.

*This patient did not fulfil SPS criteria but presented a large serrated polyp at 30 and strong CRC family history.

†This patient presented a serrated polyp count between 1 and 10.

AU, Australia; CADD, Combined Annotation Dependent Depletion Phred score; CRC, colorectal cancer; DE, Germany; gnomAD, Genome Aggregation Database variant frequency; N, no; NA, not available; NL, Netherlands; SPS, serrated polyposis syndrome; Y, yes.

To further test the association of *WNK2* with SPS predisposition, we also performed a gene-based burden test, where the aggregate burden of rare, protein-altering variants in *WNK2* was compared between our cases and control subjects. To do so, we accessed the data available from 262 healthy controls at the Collaborative Spanish Variant Server CSVS data (http://csvs.babelomics.org/).^31^ By applying the previous filters of frequency and pathogenicity, we identified two rare, protein-altering variants in this control dataset (2/262, 0.76%) and confirmed an enrichment for rare, nonsynonymous/truncating or missense variants in the *WNK2* gene in our SPS cohort (χ^2^=4.55, p value=0.03).

## Discussion

In this study, we initially analysed two independent SPS cohorts resulting in the identification of five rare, protein-altering germline variants in the *WNK2* gene. Additionally, examination of international SPS cohorts yielded 10 additional *WNK2* genetic variants in 12 SPS patients. To assess the impact on WNK2 of three of the genetic variants identified in the original cohort, we developed a cellular model using CRISPR-Cas9 technology and further performed functional assays.

WNK2 is a member of the WNK ‘With-No-Lysine(K)’ kinase subfamily, in which four different kinases have been identified (WNK1-4). This class of kinases was described to play a role in organism development and osmoregulation, but also in cancer, such as gliomas, hepatocellular carcinoma and CRC.^27 32 33^ This protein is predominantly expressed in heart, brain and colon and, unlike the other three WNKs, is not expressed in kidney.

Noteworthy, the *WNK2* gene is located at chromosomal region 9q22.31, a region previously linked to familial CRC.^34^ *WNK2* somatic mutations are found in several cancer types, including CRC, ovarian, hepatic and gastric cancer.^35 36^ *WNK2* has also been reported to be epigenetically silenced in pancreatic adenocarcinoma and gliomas.^37 38^ Interestingly, *WNK2* downregulation has also been detected in serrated polyps.^39^

WNK2 regulates the phosphorylation and activation of ERK1/2, one of the best studied MAPK cascades.^40 41^ The MAPK/ERK pathway controls many cellular processes, including cellular proliferation, cell survival, migration, invasion and adhesion. This pathway is deregulated in around one-third of all human cancers, being remarkable in CRC.^42^ Most of the alterations constitutively activate it and occur in the upstream elements of the signalling pathway, such as mutations in *BRAF* or *KRAS*.^28^ Due to its central role in many basic cellular processes, this pathway is tightly regulated at different levels of the cascade. In this sense, *WNK2* negatively regulates it by controlling the GTP-loading of Rac1.^37^ Rac1, when activated, triggers a signalling cascade resulting in the activation of PAK1, phosphorylation of the S298 residue of MEK1 and consequent activation of ERK1/2 (figure 2A).^40 41^

We functionally characterised three rare, missense germline *WNK2* variants detected in SPS cohorts including c.2105C>T (p.Pro702Leu), c.4820C>T (p.Ala1607Val) and c.6157G>A (p.Val2053Ile) by focusing on whether they altered the MAPK signalling cascade. The WNK2 knockout cellular models displayed higher phospho-PAK1/2 and phospho-ERK1/2 levels, implying that WNK2 depletion promoted the activation of the MAPK pathway and in agreement with previous results in HeLa and HT-29 cells,^40 41^ pancreatic adenocarcinoma tissue^38^ and in hepatocarcinoma cell lines.^43^ All tested *WNK2* variants showed the same tendency, with a prominent *WNK2* malfunction detected for the p.Pro702Leu variant, which displayed the most significant phosphorylation levels of both PAK1/2 and ERK1/2 in *WNK2* ^KO2^ and *WNK2* ^KO7^ clones.

We next evaluated some of the main downstream cellular processes affected by MAPK/ERK deregulation, such as cell cycle progression and cell survival. *CCND1* is an important downstream effector of the MAPK pathway and has its expression levels regulated in response to mitogenic signals.^44^ We observed that *CCND1* expression levels were increased, although not statistically significant for all *WNK2* variants, and that the activation profile was similar to the observed PAK1/2 phosphorylation levels. As multiple signalling pathways can converge on *CCND1* transcriptional activation, we hypothesise that WNK2 malfunction can influence CCND1 expression by ERK dependent and independent pathways driven by PAK1.^45^

Cell survival is also influenced by regulation of the MAPK pathway. Cells harbouring the *WNK2* variants showed an increase in their clonogenic capacity compared with WNK2-WT cells. Our results agree with previous results in hepatocellular carcinoma *WNK2-*silenced cells, in which re-expression of WNK2-WT suppressed colony formation, whereas introducing mutated WNK2 increased colony formation capacity.^43^ In addition, upregulation of *WNK2* expression has also been linked to apoptosis, senescence and autophagy in colon cancer and glioma cells, which are processes focused on cell cycle control.^46 47^

Cell adhesion is an important feature of the cell malignancy process and is closely regulated by PAK1 activation.^48^ Matrix metalloproteinases, which have been widely described as MAPK transcriptional targets, are responsible for extracellular matrix degradation and have an important role in cellular invasion processes. For this reason, we assessed both *MMP2* expression and fibronectin-mediated adhesion of our cellular models. Cells expressing tested variants showed higher *MMP2* levels than WNK2-WT cells. Moreover, the adhesion capacity of p.Pro702Leu *WNK2* variant stood out among others, suggesting a possible impact of *WNK2* impairment in extracellular matrix remodelling. Previous work in glioma cell lines had already described the negative correlation between *WNK2* and *MMP2* expression and activity, highlighting the *WNK2* importance in cell invasion and migration.^30 49^

**Figure 4:**
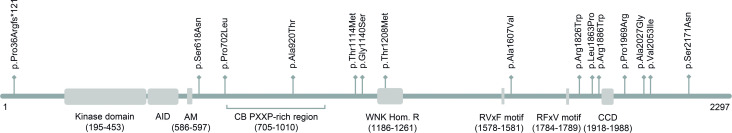
Schematic representation of WNK2 indicating the location of the identified variants. The WNK2 protein has a main kinase domain, an autoinhibitory domain (AD) and short homology regions shared with the other WNK kinases: an acidic motif (AM), the WNK homology region (WNK Hom R) and a coiled-coil domain (CCD). Some motifs and protein binding sites are also indicated, such as the compositionally biased (CB) PXXP-rich region, the RVxF motif and the RFxV motif. Additional predicted eukaryotic linear motifs (ELMs) can be found in online supplemental table 3.

WNK protein kinases have a conserved kinase domain, an autoinhibitory domain, one or two coiled-coil domains, and numerous protein interaction motifs, including PXXP proline-rich motifs and RFX/V/I motifs.^32^ Further protein motif prediction in WNK2 was performed with the eukaryotic linear motif resource to search for motifs affected by the identified genetic variants.^50^ According to this resource, a summary of the predicted effect of the 15 WNK2 genetic variants is available in online supplemental table 3, and their location in the protein structure is depicted in figure 4. Most variants are located to motifs predicted to have a functional meaning, including protein binding and phosphorylation recognition sites.^51^ Overall, the multiple protein–protein interaction motifs in WNK2 seem to reveal that, apart from its kinase activity, it could be also considered a scaffolding protein that facilitates protein–protein interactions in the MAPK cascade. In this sense, mutations in the kinase domain and those located along the *WNK2* sequence could impair its role as a MAPK regulator.

Moreover, it should be highlighted that the *WNK2* gene seems to be intolerant to loss-of-function genetic variation as evident by gene constraint scores (pLI=1, LOEUF ratio=0.12 (0.07–0.21).^52^ Together with our gene-burden test results, it would be supporting its potential role in germline predisposition to SPS.

Taking into account the number of variants found in our cohorts, the frequency of germline *WNK2* alterations in SPS could be considered ~3%. Undoubtedly, the present study is preliminary, and analysis of additional larger familial SPS cohorts and further functional studies are needed to provide more information about the prevalence and implication of germline *WNK2* mutations in SPS. As a limitation, our study used WES in the discovery cohort and alterations outside the coding sequence, in non-canonical transcripts or epimutations cannot be ruled out. Additionally, it is important to perform continued segregation analyses of the reported *WNK2* variants in the affected families to confirm (or rule out) their pathogenicity. Finally, further studies in somatic tissue of serrated lesions or CRC in *WNK2* carriers could shed light regarding mutational signatures associated with this genetic defect, as well as organoid modelling could also help to confirm the involvement of this gene in the sequence of events moving towards a serrated phenotype.

In summary, our findings indicate that germline *WNK2* variants in SPS patients may be implicated in inherited predisposition to SPS and postulate that the disruption of the role of WNK2 as a MAPK regulator could be the plausible underlying mechanism. However, a thorough assessment of the evidence for and against pathogenicity is still needed, as well as replication in additional SPS cohorts, in order to clarify a causative role for germline *WNK2* variants in SPS.

## Data Availability

Data are available on reasonable request.
