# Supplementary material for: Germline mutations in WNK2 could be associated with serrated polyposis syndrome
Source: J Med Genet. 2022 Oct 21;60(6):557–67. doi: 10.1136/jmg-2022-108684 (PMC10313964; doi:10.1136/jmg-2022-108684)
Supplement: Supplementary data [file jmg-2022-108684supp001.pdf]

SUPPLEMENTARY MATERIAL

SUPPLEMENTARY MATERIALS AND METHODS

Variant Identification

DNA extraction

Germline DNA samples were extracted using the QIAamp DNA Blood kit (Qiagen, Hilden, Germany) following the manufacturer's instructions.

WES and variant prioritization in the discovery cohort

WES was performed using the HiSeq2000 Platform (Illumina, San Diego, USA). All sequencing and quality control details were previously described.[1]

Variant prioritization

Variant prioritization was carried out considering several aspects. First, we only took into consideration those variants present in the canonical transcripts. Also, heterozygous variants were prioritized over homozygous variants since a dominant inheritance to SPS is mostly hypothesized. In addition, a minimum allele frequency of 0.1% was required for variant filtering and only nonsynonymous and/or truncating variants were prioritized. The missense variants had to fulfill at least 3 out of 6 pathogenic predictions used for analysis (PhyloP, SIFT, Polyphen, MutationTaster, CADD and LRT). The next crucial step of variant prioritization considered data integration with the first cohort results. We prioritized genes that presented germline variants in both cohorts and conducted an extensive literature research over possible connections

between candidate genes and SPS. Only candidate genes with putative pathogenic genetic variants detected in the discovery and the validation cohort were further considered. Among them, only those with a function compatible with SPS, CRC or cancer were selected.

Variant validation

Integrative Genome Viewer (IGV,http://software.broadinstitute.org/software/igv/) and Sanger sequencing (Eurofins Genomics, Luxembourg) were performed to do a final validation of the identified variants. Segregation was also carried out in patients with family history, when possible. We used the *WNK2* NM_001282394 transcript as reference.

Gene panel sequencing

Gene-panel sequencing was completed using the Illumina Miseq platform (Illumina, San Diego, USA) in germline DNA from SPS patients of the validation cohort. Library quality control was accomplished with Bioanalyzer 2000 (Agilent, Santa Clara, CA, USA). Samples were indexed with adapters containing different barcodes and pooled together, followed by massively parallel sequencing using an AmpliSeq DNA panel of 575 amplicons divided into two pools, with an amplicon length of 125-375 bp (Illumina, San Diego, USA).

Development of a cellular model for variant characterization

Cell lines

HT-29 (Cat No. HTB-38) and HEK293T (Cat No. CRL-3216) cell lines were purchased from American Type Culture Collection (ATCC, Manassas, VA). HT-

Soares de Lima Y, *et al. J Med Genet* 2023; 60:557–567. doi: 10.1136/jmg-2022-108684

29 was cultured with McCoy 5A (Modified) (Gibco, Waltham, MA) and the later with DMEM (Gibco), both supplemented with 10% fetal bovine serum (FBS) at 37ºC in 5%CO_2. All functional assays were performed with medium supplemented with 5% FBS.

WNK2 knock-out generation

The CRISPR-Cas9 system was used to perform the *WNK2* knock-out. The sgRNA was designed using The Benchling CRISPR guide design tool (http://benchling.com). Bottom and top strands of sgRNA were purchased from IDT (Coralville, IA) and cloned into the BbsI site of the PX458 plasmid following the protocol by Ran F. A. et al.[2] The plasmid was transiently transfected into HT-29 cells using X-tremeGENE HP DNA transfection reagent (Roche, Basel, Switzerland). After 3-5 days, cells were sorted for GFP+ cells. Sorted cells were let to rest for 2 days and then seeded at 1 cell/well density in 96-well plates with HT-29 conditioned media. After two weeks, several clones were analyzed for *WNK2* gene editing. DNA, RNA and protein samples were extracted and analyzed. Genomic DNA was analyzed by Sanger sequencing (Eurofins Genomics) and screened for indel insertions manually and by using the TIDE web tool (https://tide.nki.nl/).[3] Predicted exonic off-target regions with high homology with the target region were identified by using the CRISPOR tool (http://crispor.tefor.net/) and excluded by PCR and Sanger sequencing.[4] Gene expression was accessed by quantitative real-time PCR, and protein expression was evaluated by Western-Blot.

Site-directed mutagenesis

To generate *WNK2* variants by site-directed mutagenesis, the pUC57-*WNK2* vector (**supplementary table 1**) was digested into smaller segments because of the length of *WNK2* and polymerase restrictions. We used three different restriction enzymes: XbaI, NcoI and SalI. Both 4083 bp (5' XbaI-3' NcoI) and 2868 bp (5' NcoI-3' SalI) fragments, which were part of the coding sequence of *WNK2*, were recovered and subcloned separately into the pUC19 plasmid. The generation of *WNK2* variants was carried out using the Q5 Site-Directed Mutagenesis Kit (NEB, Ipswich, MA) according to the manufacturer's instructions. Primers were designed using the NEBaseChanger tool and purchased from IDT (Coralville, IA). Mutated fragments were inserted back into the pUC57-WNK2 backbone. Variant insertion was verified by Sanger sequencing.

Variant reintroduction into the *WNK2* knock-out model

To achieve a stable, reproducible *WNK2* expression, the *WNK2* ORF was transferred from pUC57 into the lentiviral plasmid pLVX-TetOne-Puro (TakaraBio, Kusatsu, Japan). Each mutated *WNK2* ORF with its variant and the *WNK2* WT sequence were cloned independently in the pLVX-TetOne-Puro vector thanks to the additional EcoRI and AgeI restriction sites flanking the *WNK2* ORF.

Lentiviral particles were generated for each *WNK2* variant. Briefly, HEK293T cells were co-transfected with the lentiviral vectors (VSVG2, psPAX2) and pLVX-TetOne-Puro containing either WNK2 ORF WT or the mutated WNK2 ORF using the CalPhos Mammalian Transfection kit (TakaraBio, Kusatsu, Japan). The supernatant was recovered both after 24h and 48h after

Soares de Lima Y, *et al*. *J Med Genet* 2023; 60:557–567. doi: 10.1136/jmg-2022-108684

transfection. Viral particles were pooled and then concentrated in a 10% sucrose cushion at 20,000x*g* for 3 hours at 4ºC. The pellet was resuspended in 5mL of McCoy medium and prepared for infection. HT-29 cells were plated at 600,000 cells/well in a 6-well plate and let grow for two days before infection. Lentiviral transduction was performed in the presence of 8 μg/mL of polybrene. The plate was centrifuged at 1600 x*g* for 2h at 32 ºC and then, culture media was removed and replaced with fresh complete media.

Functional titer by limiting dilution was performed to establish an optimal multiplicity of infection (MOI) of 1, which was further validated by Real-Time PCR. After obtaining genomic DNA samples from infected cells, the viral *WPRE* region was amplified by RT-PCR with RealQ Plus 2x Master Mix (Ampliqon, Odense, Denmark) and specific primers. The assessment of lentiviral vector copies was inferred from a standard curve using known amounts of pLVX-TetOne. The final integrated lentivirus copy number was calculated as total viral copies/number of cells assayed. The number of cells was estimated from the amount of genomic DNA loaded in the RT-PCR, considering that the mass of the human genome (haploid) is 3,181 pg.

<u>Protein extraction and Western Blot</u>

To obtain whole-cell protein extracts, cells were lysed with RIPA buffer solution (Sigma-Aldrich, MA, USA) supplemented with cOmplete Protease Inhibitor Cocktail and PhosSTOP (Roche, Basel, Switzerland) and recovered using a cell-scrapper. Protein concentration was measured using Pierce BCA Protein Assay kit (ThermoFisher, Waltham, MA). Western blot for WNK2 detection was carried out running 40ug of protein extract of each sample in NuPAGE™ 3-8%

Tris-Acetate gels, according to the manufacturer's protocol (ThermoFisher, Waltham, MA). Protein transfer into PVDF membranes (Millipore, Bedford, MA) was left to perform overnight at 4ºC. Proteins were blotted with anti-WNK2 primary antibody an anti-rabbit secondary Dylight 800 antibody. Protein detection was carried out using Odyssey Imaging System (LI-COR, Linconl, NE).

RNA extraction and Quantitative Real-time PCR

Total RNA was extracted using the RNeasyMini Kit according to the manufacturer's protocol (Qiagen). RNA was quantified using Nanodrop (ThermoFisher, Waltham, MA) and retrotranscribed with the High-Capacity cDNA reverse Transcription kit (Applied Biosystems). Quantitative PCR was performed in the QuantStudio 1 System (Applied Biosystems) by using Taqman probes for *WNK2*, *CCDN1* and *GAPDH* as a housekeeping gene reference. *MMP2* gene expression was measured by using RealQ Plus 2x Master Mix (Ampliqon, Odense, Denmark) and specific primers for *MMP2* and *GAPDH* detection. The relative quantification was performed with the –ΔΔCt method.

Functional Characterization of Genetic Variants

MAPK pathway activity: ERK1/2

To detect total phospho-ERK1/2, 600,000 cells were plated in a P60 dish and cultured in McCoy 5A media supplemented with 5% FBS and 1µg/mL of doxycycline for two days. After 24h starvation, cells were induced with 1 ng/mL hEGF (Miltenyi Biotec, Bergisch Gladbach, Germany) for 10 minutes. To preserve phosphorylated proteins, cells were lysed using Lysis buffer 6 (Bio-

Techne, Minnesota, USA) supplemented with Pepstatin A, Leupeptin and Aprotinin (10µg/mL) (Merck, Darmstadt, Germany) and recovered using a cell-scrapper. The whole-cell extract was incubated for 15 minutes on ice and centrifuged at 2000x*g* for 5 minutes at 4ºC. Total protein extract was quantified using Pierce BCA Protein Assay kit (ThermoFisher Waltham, MA), and 15 ug of total protein extracts were assayed. Detection of phosphorylated ERK1/2 was performed using the Phospho-ERK1 (T202/Y204)/ERK2 (T185/Y187) DuoSet IC ELISA kit according to the manufacturer's protocol (Bio-Techne, Minnesota, USA). Phospho-ERK1/2 levels between EGF-stimulated and non-stimulated conditions were quantified.

<u>MAPK pathway activity: PAK1/2 phosphorylation</u>

To detect phosphorylated PAK1, an In-Cell ELISA (ICE) assay was performed. 96-well plates were coated with 50 µg/mL Poly-L-Lysine before cell seeding. 25,000 cells were plated per well and cultured in McCoy 5A media supplemented with 5% FBS and 1µg/mL of doxycycline. After 48 hours, cells were stimulated with 10 ng/mL of hEGF for 5 minutes. To immediately fix phosphorylated proteins and avoid cell detachment, cells were treated with a 2X 8% formaldehyde solution, supplemented with 100 mg/L $Ca^{2+/}Mg^{2+}$ and PhosSTOP for 20 min. After fixation, cells were permeabilized with 0.05% Triton X100 for 20 min and blocked with the Intercept Blocking Buffer (LI-COR, Lincoln, NE) for 1h. Cells were incubated with both phospho-PAK1 (rabbit) and β-actin (mouse) antibodies overnight at 4 ºC. The multiplexed detection of both targets was performed with the anti-mouse Dylight 800 (ThermoFisher) and anti-rabbit IRDye 680RD (LICOR) antibodies. Plates were scanned with

Soares de Lima Y, *et al. J Med Genet* 2023; 60:557–567. doi: 10.1136/jmg-2022-108684

Odyssey (LI-COR) and analysed with Image Studio 4.0 software. Phospho-PAK1 levels between EGF-stimulated and non-stimulated conditions were quantified.

References

1. Soares de Lima Y, Arnau-Collell C, Díaz-Gay M, Bonjoch L, Franch-Expósito S, Muñoz J, Moreira L, Ocaña T, Cuatrecasas M, Herrera-Pariente C, Carballal S, Moreno L, Díaz de Bustamante A, Castells A, Bujanda L, Cubiella J, Rodríguez-Alcalde D, Balaguer F, Castellví-Bel S. Germline and Somatic Whole-exome Sequencing Identifies New Candidate Genes Involved in Familial Predisposition to Serrated Polyposis Syndrome. *Cancers* 2021;13(4):1–21.
2. Ran FA, Hsu PD, Wright J, Agarwala V, Scott DA, Zhang F. Genome engineering using the CRISPR-Cas9 system. *Nat Protoc* 2013;8(11):2281–2308.
3. Brinkman EK, Chen T, Amendola M, van Steensel B. Easy quantitative assessment of genome editing by sequence trace decomposition. Nucleic Acids Res 2014; 42(22):e168–e168.
4. Concordet JP, Haeussler M. CRISPOR: Intuitive guide selection for CRISPR/Cas9 genome editing experiments and screens. *Nucleic Acids Res* 2018;46(W1):W242-W245.

Supplementary table 1. List of reagents (plasmids, restriction enzymes and Taqman probes) used in this study.

Reagent	Source	Identifier
**Plasmids**		
pSpCas9(BB)-2A-GFP (PX458)	Addgene (gift from Feng Zhang)	Cat No. 48138
pUC19	Invitrogen (Waltham, MA)	Cat No. 18265017
pLVX-TetOne-Puro inducible system	TakaraBio, provided by Dr. Eulàlia de Nadal (IRB, Barcelona)	Cat No. 631847
Second generation packaging vectors pCMV-VSV-G2 and psPAX2	provided by Dr. Ester Sánchez-Tilló (IDIBAPS, Barcelona)	
WNK2 custom gene synthesis (pUC57-*WNK2*)	Bio Basic Inc. (Toronto, Canada).	
**Restriction enzymes**		
BbsI	New England Biolabs (Ipswich, MA)	Cat No. R0539S
NcoI-HF	New England Biolabs (Ipswich, MA)	Cat No. R3193S
SalI-HF	New England Biolabs (Ipswich, MA)	Cat No. R3138S
EcoRI-HF	New England Biolabs (Ipswich, MA)	Cat No. R3101S
AgeI-HF	New England Biolabs (Ipswich, MA)	Cat No. R3552S
**Antibodies**		
rabbit polyclonal anti-WNK2	Invitrogen (Waltham, MA)	Cat No. PA5-53440
rabbit monoclonal anti-GAPDH (clone 14C10)	Cell Signaling (Danvers, MA)	Cat No. 2118
rabbit monoclonal anti-vinculin (7F9)	Santa Cruz Biotechnology	Cat No. sc-73614
rabbit polyclonal phospho-PAK1(Ser199/204)/PAK2 (Ser192/197)	Cell Signaling (Danvers, MA)	Cat No. 2605
mouse monoclonal anti-β-actin	Sigma Aldrich (St Louis, MO)	Cat No. A2228
DyLight 800 goat anti-mouse IgG	Invitrogen (Waltham, MA)	Cat No. SA5-10176
IRDye 680RD goat anti-rabbit IgG	LI-COR (Lincoln, NE)	Cat No. 926-68071

Soares de Lima Y, *et al. J Med Genet* 2023; 60:557–567. doi: 10.1136/jmg-2022-108684

DyLight 800 goat anti-rabbit IgG	Invitrogen (Waltham, MA)	Cat No. SA5-10036
**Taqman probes**		
GAPDH-VIC/MGB	Applied Biosystems (Foster City, CA)	4326317E
WNK2-FAM/MGB	Applied Biosystems (Foster City, CA)	hs00396601_m1
CCND1-FAM/MGB	Applied Biosystems (Foster City, CA)	hs00765553_m1

Supplementary table 2. List of primers used in this study.

	Forward	Reverse
**Validation cohort validation**		
WNK2 *c*.2105C>T	5’-AGAGCTGCTGCTGAGTGTG-3’	5’-ACAGTGGCCACAGGAGGT-3’
WNK2 c.3341C>T	5’-CCAGACTGCCACACTTCTG-3’	5’-CTCTAGGACTAAGACCCCAGG-3’
WNK2 c.5588T>C	5’-AGCGACTCTGGGGACGAG-3’	5’-CATTGTCGCTGCTGATGTAG-3’
**WNK2 knock-out generation**		
WNK2 sgRNA	5’-caccGTCGCCGGGACGAGCCCGAAG-3’	5’-aaacCTTCGGGCTCGTCCCGGCGAC-3’
WNK2 sgRNA cut validation	5’-GCTTCTGCTCTGCAAGACG-3’	5’-CAGAGGGTGACACACATGG-3’
**Off-target validation**		
CENPB	5’-GAGCCGCTTTGTCTCGGG-3’	5’-TGCGCAGGTCCGGATTCTC-3’
MTX1	5’-GAGTTCCCGTCACCTAAGCG-3’	5’-CTCTTGCCAAACTGCACGTG-3’
ZNF865	5’-CTTTCCGATCCTCCACGCC-3’	5’-CTGACTTGCTAGGGGTTGGG-3’
**Site directed mutagenesis**		
WNK2 c.2105C>T	5’-TTCCCGGATCtGGCCATGAGC-3’	5’-GTGCTGCTGGAGGGACGG-3’
WNK2 c.4820C>T	5’-ACCTCCTCAGtCTCAGCTGGG-3’	5’-GGGCACGTGCTCCTGGTA-3’
WNK2 c.6157G>A	5’-GCCCTGCTCCaTCCGGGCCTC-3’	5’-TGCTGGGTCTGCACTGCCTTC-3’
c.2105C>T validation	5’-TCTACCGTGTACTCAGACTCG-3’	5’-ATCTGGAGGGGCTTCAGC-3’
c.4820C>T validation	5’-GTGGACAGCACCATCAAGA-3’	5’-CCTGAGACCGCCTCCTTA-3’
c.6157G>A validation	5’-CAAGCTGCTAAATCCCCTGG-3’	5’-ATAGGTCACTCTCTCAGACGTT-3’
**Real-Time PCR**		

Soares de Lima Y, *et al. J Med Genet* 2023; 60:557–567. doi: 10.1136/jmg-2022-108684

WPRE	5'-CCGTTGTCAGGCAACGTG-3'	5'-AGCTGACAGGTGGTGGCAAT-3'
MMP2	5'-AGCGAGTGGATGCCGCCTTTAA-3'	5'-CATTCCAGGCATCTGCGATGAG-3'
GAPDH	5'-GTCTCCTCTGACTTCAACAGCG-3'	5'-ACCACCCTGTTGCTGTAGCCAA-3'

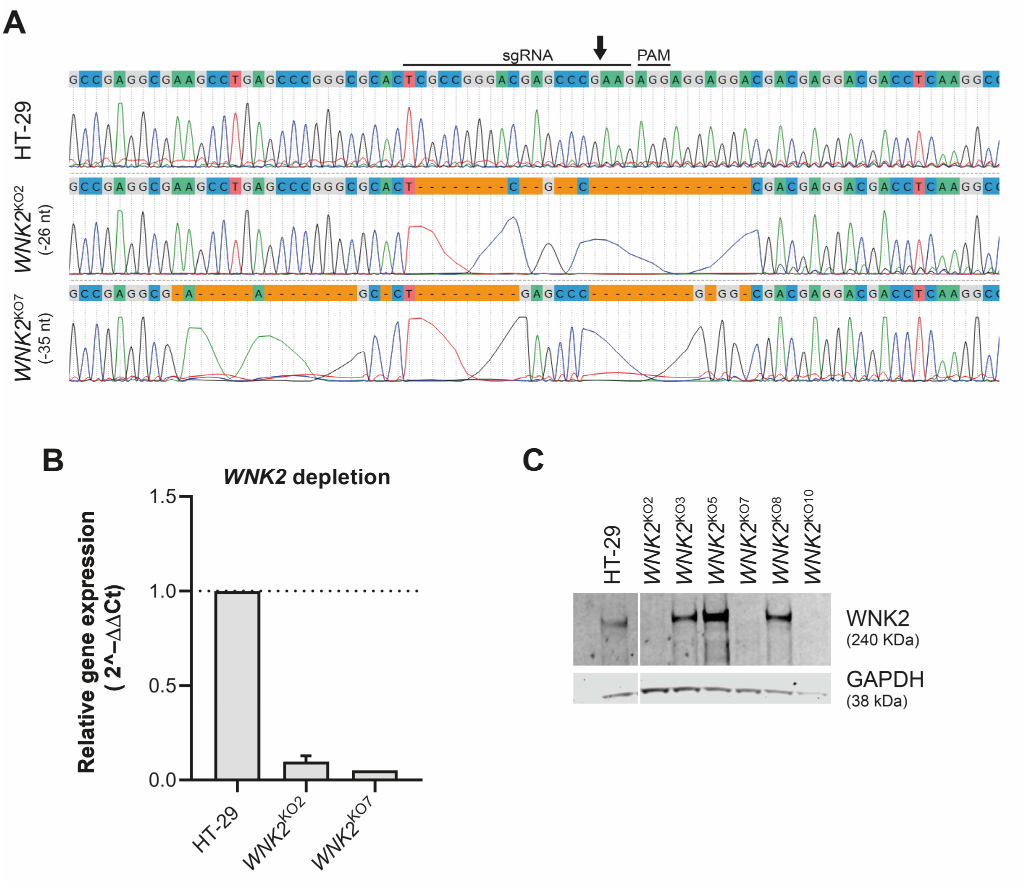

Supplementary figure 1. CRISPR/Cas9-mediated *WNK2* gene inactivation in HT-29 cells. (A) Sanger sequencing profile of both clones aligned with the wild-type sequence, showing indel mutations near the break site (arrow). The 20-nt sgRNA and the 3-nt PAM sequences are depicted. (B) *WNK2* mRNA relative expression of both clones assessed by Real Time PCR. Data represent mean ± SD (n = 2). The experiment was performed in triplicate and repeated two times (n = 2). (C) Representative Western Blot analysis of several *WNK2*^{KO} clones, confirming *WNK2* depletion on clones *WNK2*^{KO2} and *WNK2*^{KO7}. Uncropped Western can be consulted further down in this same document.

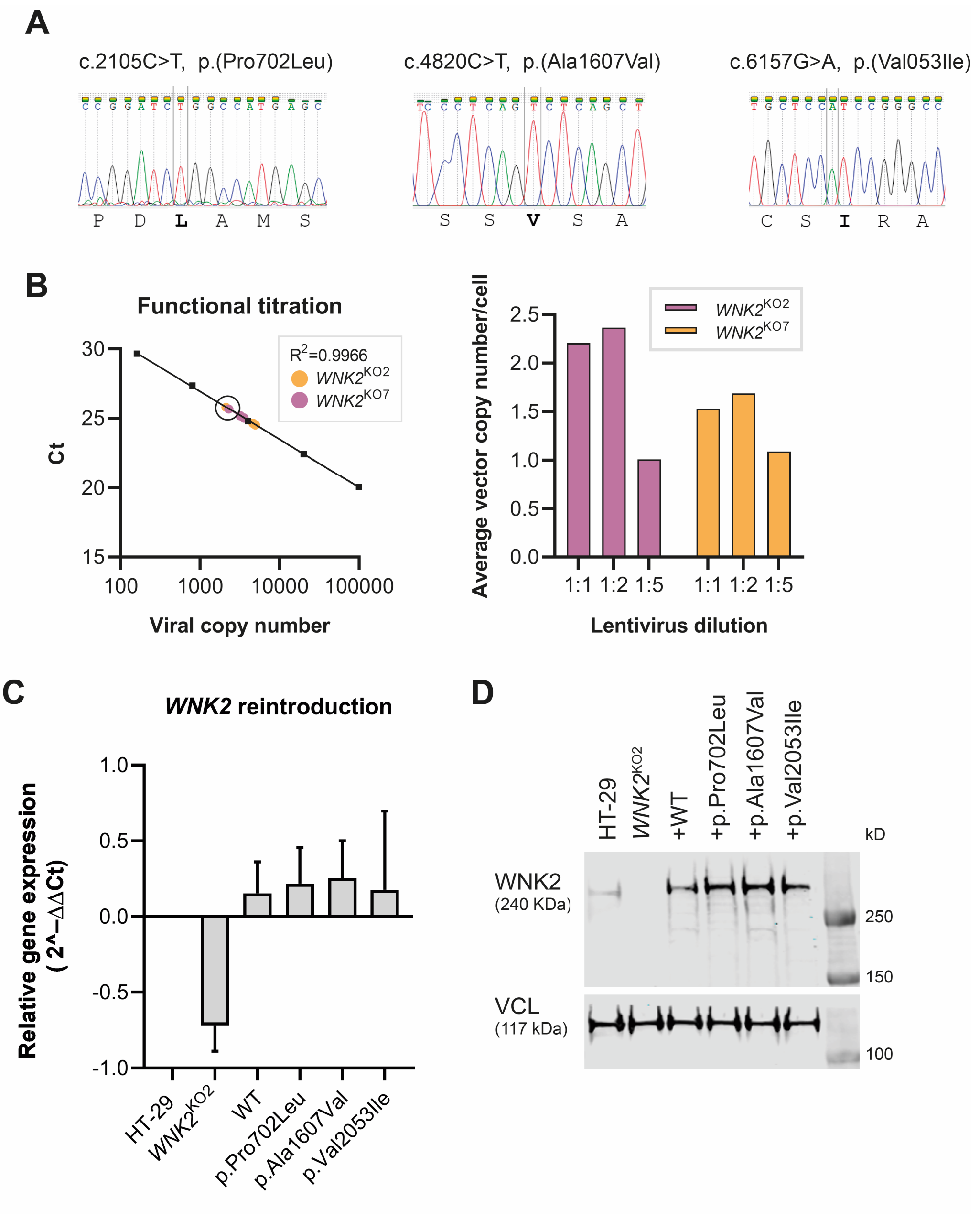

Supplementary figure 2. Development of a cellular model for *WNK2* variant characterization. (A) Sanger sequencing of site-directed mutagenesis products confirmed the correct generation of WNK2 p.(Pro702Leu), p.(Ala1607Val) and p.(Val2053Ile) variants. Changes in the nucleotide sequence are marked, and the altered amino acid sequence is highlighted in bold. (B) Representative functional titration of the lentiviral particles stock to ensure that a single lentiviral

copy is inserted per cell. The example corresponds to the pLVX-TetOne-WNK2-WT plasmid reintroduced into both clones to rescue the expression of wild-type *WNK2*. On the left panel, the WPRE lentiviral sequence is detected by SYBR green-based Real-Time PCR by using a standard curve with known lentiviral vector copy numbers. Samples infected with different dilutions of the lentiviral particles stock were assayed, and the selected dilution (one vector copy number/cell) is circled. On the right panel, the extrapolated viral copy numbers were corrected taking into account the amount of DNA assayed, determining the integrated viral copies per cell. Samples were assayed in triplicate. (C) *WNK2* mRNA relative expression in *WNK2*^{KO2} cells expressing either the WT sequence (rescued phenotype) or each of the selected variants. The dose of 1 ug/mL of doxycycline induced near wild-type *WNK2* expression levels. Data represent mean ± SD The experiment was performed in triplicate and repeated two times (n = 2). (D) Representative Western Blot analysis of *WNK2*^{KO2} cells expressing either the WT sequence or each of the selected variants after 1 ug/mL doxycycline treatment. VCL, vinculin.

Soares de Lima Y, *et al. J Med Genet* 2023; 60:557–567. doi: 10.1136/jmg-2022-108684

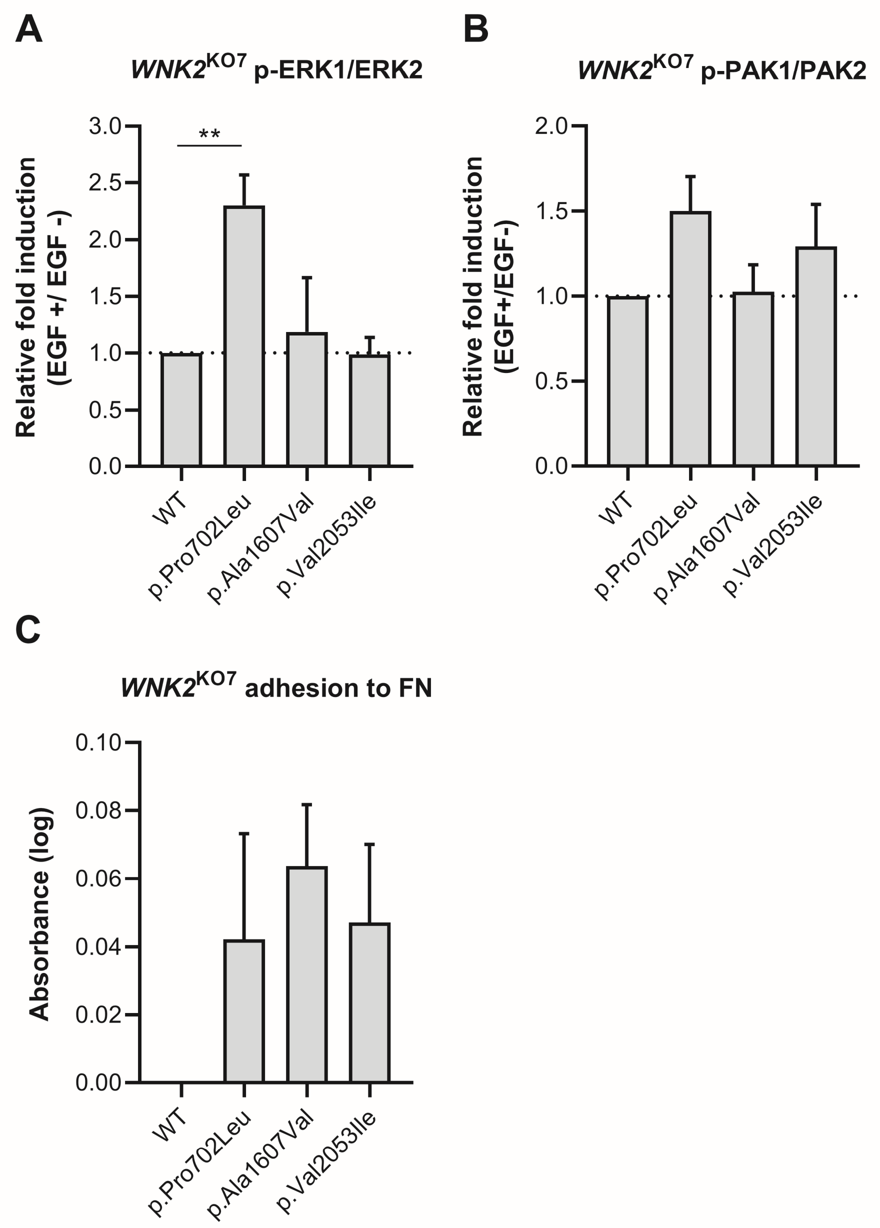

Supplementary figure 3. Functional characterization of *WNK2*^{KO7} cellular model. *WNK2*^{KO7} cells expressing either the WT sequence (rescued phenotype) or each of the selected variants were functionally characterized. Data is normalized against the observed values in *WNK2* WT. (A) ERK1/2 phosphorylation levels measured by ELISA in cell samples treated or untreated with 1 ng/mL hEGF. Data is displayed as EGF+/- ratio (n = 3; mean ± SD). (B)

Soares de Lima Y, *et al. J Med Genet* 2023; 60:557–567. doi: 10.1136/jmg-2022-108684

PAK1/2 phosphorylation levels measured by an In-Cell ELISA (ICE) assay in cell samples treated or untreated with 10 ng/mL hEGF. Data is displayed as EGF+/- ratio (n = 3; mean ± SD) (C) Cells were cultured on fibronectin (FN)-coated plates for 1 hour and the adherent cells were detected by crystal violet staining (mean ± SD) The experiments were performed in triplicate and repeated three times (n = 3). **P<.01, ANOVA with Fisher's LSD post hoc test.

Soares de Lima Y, *et al. J Med Genet* 2023; 60:557–567. doi: 10.1136/jmg-2022-108684

Supplementary table 3. Predictions according to the eukaryotic linear motif (ELM) resource for the *WNK2* genetic variants identified in additional external SPS cohorts. ELM is a repository of manually curated experimentally validated short linear protein motifs and it is used for investigating functional regions in proteins.

Genetic variant	Exon	Cohort	ELM domain	Positions	Instances (matched sequence)	ELM description
c.106_107insG (p.Pro36Argfs*121)	1	DE	Frameshift			
c.1853G>A (p.Ser618Asn)	8	NL	DEG_SCF_TRCP1_1	617-623	DSGQGST	The DSGxxS phospho-dependent degron binds the F box protein of the SCF-betaTrCP1 complex. The degron is found in various proteins that function in regulation of cell state
c.2105C>T (p.Pro702Leu)	9	ES	DEG_SCF_FBW7_1	712-719	PPSTPMPT	The TPxxS phospho-dependent degron binds the FBW7 F box proteins of the SCF (Skp1_Cullin-Fbox) complex.
			LIG_SH3_3	705-711	SFAPVLP	This is the motif recognized by those SH3 domains with a non-canonical class I recognition specificity
c.2758G>A (p.Ala920Thr)	11	AU	DOC_WW_Pin1_4	924-929	VPPSPH	The Class IV WW domain interaction motif is recognised primarily by the Pin1 phosphorylation-dependent prolyl isomerase

Soares de Lima Y, *et al. J Med Genet* 2023; 60:557–567. doi: 10.1136/jmg-2022-108684

			LIG_SH3_3	922-928	TDVPPSP	This is the motif recognized by those SH3 domains with a non-canonical class I recognition specificity
			MOD_GSK3_1	924-931	VPPSPHHT	GSK3 phosphorylation recognition site
			MOD_ProDKin_1	924-930	VPPSPHH	Proline-Directed Kinase (e.g. MAPK) phosphorylation site in higher eukaryotes
c.3341C>T (p.Thr1114Met)	12	ES	MOD_GSK3_1	1107-1114	PCPTVQLT	GSK3 phosphorylation recognition site
c.3418G>A (p.Gly1140Ser)	14	DE	MOD_CK1_1	1134-1140	SCESYGG	CK1 phosphorylation site
				1141-1147	SDVTSGK	CK1 phosphorylation site
			MOD_CK2_1	1142-1148	DVTSGKE	Casein kinase 2 (CK2) phosphorylation site
			MOD_GSK3_1	1134-1141	SCESYGGS	GSK3 phosphorylation recognition site
			MOD_GSK3_1	1138-1145	YGGSDVTS	GSK3 phosphorylation recognition site
c.3623C>T (p.Thr1208Met)	15	DE	DOC_PP1_RVXF_1	1210-1217	NHKMVTFK	Protein phosphatase 1 catalytic subunit (PP1c) interacting motif binds targeting proteins that dock to the substrate for dephosphorylation. The motif defined is [RK]{0,1}[VI][^P][FW]
c.4820C>T	20	ES	MOD_CK1_1	1605-1611	SSASAGT	CK1 phosphorylation site
			MOD_GSK3_1	1608-1614	HVPTSSAS	GSK3 phosphorylation recognition site
			DOC_WW_Pin1_4	1608-1613	SAGTPV	Pin1 phosphorylation-dependent

Soares de Lima Y, *et al. J Med Genet* 2023; 60:557–567. doi: 10.1136/jmg-2022-108684

(p.Ala1607Val)						prolyl isomerase recognition site
c.5476C>T (p.Arg1826Trp)	23	DE	LIG_14-3-3_CanoR_1	1826-1836	RRAQTASSIEV	Canonical Arg-containing phospho-motif mediating a strong interaction with 14-3-3 proteins
c.5588T>C (p.Leu1863Pro)	23	ES	MOD_GSK3_1	1859-1866	KQASLPVS	GSK3 phosphorylation recognition site
c.5656C>T (p.Arg1886Trp)	23	DE, NL	MOD_GSK3_1	1882-1889	QRPSRAGS	GSK3 phosphorylation recognition site
			MOD_PKA_2	1882-1888	QRPSRAG	Secondary preference for PKA-type AGC kinase phosphorylation
c.5906C>G (p.Pro1969Arg)	24	AU	LIG_SH3_3	1963-1969	LGKPLPP	This is the motif recognized by those SH3 domains with a non-canonical class I recognition specificity
			DOC_MAPK_DCC_7	1965-1973	KPLPPNVGF	A kinase docking motif mediating interaction towards the ERK1/2 and p38 subfamilies of MAP kinases
			DOC_MAPK_MEF2A_6	1965-1973	KPLPPNVGF	A kinase docking motif that mediates interaction towards the ERK1/2 and p38 subfamilies of MAP kinases
c.6080C>G (p.Ala2027Gly)	25	DE	MOD_GSK3_1	2027-2034	AQASVGLT	GSK3 phosphorylation recognition site
c.6157G>A (p.Val2053Ile)	25	ES	LIG_14-3-3_CanoR_1	2054-2058	RASLS	Canonical Arg-containing phospho-motif mediating a strong interaction with 14-3-3

Soares de Lima Y, *et al. J Med Genet* 2023; 60:557–567. doi: 10.1136/jmg-2022-108684

						proteins.
c.6512G>A (p.Ser2171Asn)	28	DE	NA	NA	NA	NA

ELM Eukaryotic Linear Motif, NA, not available. DE, Germany; NL, Netherlands; ES, Spain; AU, Australia.

Soares de Lima Y, *et al. J Med Genet* 2023; 60:557–567. doi: 10.1136/jmg-2022-108684

Uncropped Western Blot – Supplementary figure 1C

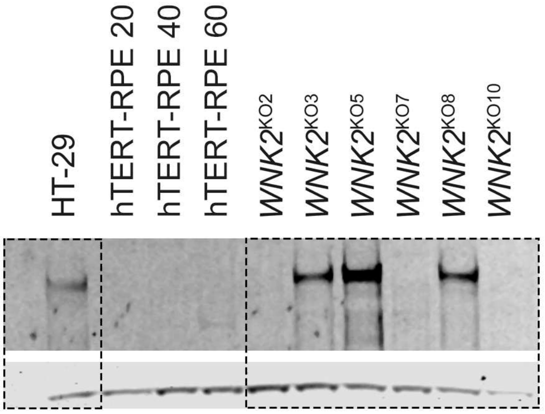

Soares de Lima Y, *et al. J Med Genet* 2023; 60:557–567. doi: 10.1136/jmg-2022-108684
